# Evaluation of Publicly Financed and Privately Delivered Model of Emergency Referral Services for Maternal and Child Health Care in India

**DOI:** 10.1371/journal.pone.0109911

**Published:** 2014-10-31

**Authors:** Shankar Prinja, Pankaj Bahuguna, P. V. M. Lakshmi, Tushar Mokashi, Arun Kumar Aggarwal, Manmeet Kaur, K. Rahul Reddy, Rajesh Kumar

**Affiliations:** 1 School of Public Health, Post Graduate Institute of Medical Education and Research, Chandigarh, India; 2 National Health Systems Resource Centre, Ministry of Health and Family Welfare, New Delhi, India; Monash University, Australia

## Abstract

**Background:**

Emergency referral services (ERS) are being strengthened in India to improve access for institutional delivery. We evaluated a publicly financed and privately delivered model of ERS in Punjab state, India, to assess its extent and pattern of utilization, impact on institutional delivery, quality and unit cost.

**Methods:**

Data for almost 0.4 million calls received from April 2012 to March 2013 was analysed to assess the extent and pattern of utilization. Segmented linear regression was used to analyse month-wise data on number of institutional deliveries in public sector health facilities from 2008 to 2013. We inspected ambulances in 2 districts against the Basic Life Support (BLS) standards. Timeliness of ERS was assessed for determining quality. Finally, we computed economic cost of implementing ERS from a health system perspective.

**Results:**

On an average, an ambulance transported 3–4 patients per day. Poor and those farther away from the health facility had a higher likelihood of using the ambulance. Although the ERS had an abrupt positive effect on increasing the institutional deliveries in the unadjusted model, there was no effect on institutional delivery after adjustment for autocorrelation. Cost of operating the ambulance service was INR 1361 (USD 22.7) per patient transported or INR 21 (USD 0.35) per km travelled.

**Conclusion:**

Emergency referral services in Punjab did not result in a significant change in public sector institutional deliveries. This could be due to high baseline coverage of institutional delivery and low barriers to physical access. Choice of interventions for reduction in Maternal Mortality Ratio (MMR) should be context-specific to have high value for resources spent. The ERS in Punjab needs improvement in terms of quality and reduction of cost to health system.

## Introduction

Institutional intra-partum care is an effective strategy to reduce the maternal and infant mortality [1]. A significant increase in institutional deliveries was reported with the *Janani Suraksha Yojana* (JSY) – conditional cash transfer scheme, which started in 2005 [2]–[4]. Maternal mortality ratio (MMR) and infant mortality rate (IMR) also declined during the same period, though causal relationship is not proven empirically [5]–[7]. Notwithstanding increase in institutional deliveries during the last decade in India, physical and financial barriers to access institutional intra-partum care were reported in recent coverage evaluation surveys [8].

Strengthening emergency referral services (ERS) to improve the access for facility-based intrapartum care is a major initiative of Government of India [9]. A variety of models have been used for implementing the service depending upon health infrastructure and various other factors in different states. Though the ERS implemented across all states is publicly financed but the delivery mechanism varies. In states such as Jharkhand, Gujarat, Madhya Pradesh and Haryana, ERS is publicly delivered. In contrast, states like Bihar, Kerala, Rajasthan and Punjab are providing ERS in public-private partnership (PPP) mode with public funding and private delivery [10].

In an earlier evaluation of Haryana model of referral transport system we reported a positive impact of referral transport service on institutional delivery in the state at a cost of INR 562 (USD 9.4) per patient transported [11], [12]. Further, in Haryana, the publicly financed and publicly delivered model lead to the equitable utilization of the institutional service for delivery care.

A preliminary national assessment of referral transport service in India reported that privately delivered referral services are much costlier than publicly delivered service [10]. Apart from high cost, there are operational and management issues. However, there is no effectiveness assessment of PPP model of referral services in enhancing public sector health care utilization.

Hence, we undertook a comprehensive evaluation of referral services in Punjab state of India, to assess its extent and pattern of utilization, and impact on public sector institutional deliveries. Secondly, we also assessed its quality and cost in Punjab state.

## Methodology

### Study Setting

Punjab is the 15^th^ most populous state in India, with 22 districts. It has an overall population of 27 million, 13.7% growth rate and 73% literacy rate [13]. The state MMR of 155 (95% confidence interval; 85,226) per 100,000 live births is below the national average of 178 [7]. ERS service was launched in Punjab on 3^rd^ April, 2011 under public-private partnership with public financing and private delivery. A state-level 24×7 call centre and office of the private provider was established at Amritsar district. Aim of ERS was to provide free transport service to pregnant women, neonates, post natal cases, infants and children with ill-health, victims of road side accident and for all other health emergencies in the general population. ERS in Punjab was implemented in four different phases. Out of 240 ambulances, 90 ambulance were launched in the first phase (April 2011) which were stationed in 12 districts, 50 ambulances in 18 districts in second phase (July 2011) and another 50 ambulances serving all 20 districts in third phase (August and September 2011). Finally, 50 more ambulances were added in fourth phase (October and November 2011). Distribution of vehicles was based on a criterion of 10 ambulances per million population. Out of the 22 districts in Punjab, 2 districts were created out of existing districts after the introduction of ERS system in Punjab. These two new districts were part of their parent districts in the Management Information System (MIS) of the service provider. Hence we report results for 20 districts.

The ambulances were stationed in the district at district hospital, community health centres and primary health centres. Ambulance could be called to place of emergency by dialling a toll-free number ‘108’ in any district throughout the state. Nearest ambulance available is dispatched by call centre operator using a well-functioning geographic information system (GIS). ERS is primarily limited for transportation to public sector institutions except if there is strong insistence by beneficiary/attendants for private facility.

### Data Collection: Source and Methods

Both primary and secondary data was collected. Secondary data for almost 0.4 million calls received during one year period from 1^st^ April, 2012 to 31^st^ March, 2013 for all the 20 districts of Punjab, as recorded in database of 108 service provider was obtained. This data was drawn from the ERS tracking software which is generated from the calls received at centralized call-centre for availing ERS. The response to the call, i.e. whether service was availed or declined was also fed into the software by call centre operator. The information captured in this data source comprised of time taken to respond to emergency, type of health care provider, i.e. public or private, level of service provider etc. Month-wise and district-wise data on public sector institutional deliveries in Punjab from the year 2008 to 2013 was obtained from Punjab Heath System Corporation (PHSC).

Primary data on users and non-users of 108 referral service was collected at facility level in three districts i.e. Amritsar, Roopnagar and Sangrur of Punjab. Users were those patients who reported in the health facility, having utilized 108 ERS service; while ‘non-users’ were those who reached the health facility by any other means of transport. Choice of selection of these three districts was primarily based on the performance of ERS, which was determined by number of calls per ambulance per day or calls per ambulance per million population. As there was not much diversity geographically therefore the level of infrastructure in the district was also kept under the view while selecting the districts. Sangrur had lowest rate of utilization, Roopnagar - medium utilization, and Amritsar was randomly chosen from among group of high performing districts. Health facilities within the district were selected randomly. In the health facility, consecutive sampling was used for selection of users and non-users. All patients who reported during the period of data collection were included in the study. Patients were interviewed using pre-tested semi-structured to collect data on basic socio-demographic characteristics, reasons for using and not using 108, client satisfaction, level of severity etc. among users and non-users. We recruited a total of 411 users and 999 non-users of 108 referral transport were recruited in the present study.

The quality of 108 ambulance service in Punjab was evaluated using a checklist designed by Delhi Government as a standard for a Basic Life support (BLS) ambulance [14]. All the ambulances in 21 randomly selected health facilities in Amritsar and Sangrur district were inspected against this checklist to assess their adequacy in terms of infrastructure, design, availability of drugs, consumables and other life support systems besides trained technicians and manpower.

We estimated the economic cost of implementing referral service in Punjab from the health system's perspective. Cost data for the 108 ERS was collected from the accounts department of the PHSC. Non-recurrent or capital costs and recurrent or operational costs were elicited. The non-recurrent costs comprises of cost of 240 vehicles, set up cost of emergency response centre in district Amritsar, information-technology infrastructure (including software) and pre-operational costs i.e. trainings, recruitments, marketing, administration and communication costs. Operational costs were the annual costs paid to private provider for delivering the service, which was fixed per ambulance, subject to a minimum of 3 calls per day per ambulance.

### Data Analysis

#### Extent and Pattern

Data of 391369 calls, received from 1^st^ April, 2012 to 31^st^ March, 2013 was analyzed to assess the extent and pattern of utilization. Utilization pattern was assessed by the gender, medical condition of the patient and type of health facility. To ascertain the impact of 108 referral transport service on equitable utilization of public health facilities, we analysed the primary data on users and non-users of 108 using multiple logistic regression.

#### Quality of Service

To evaluate the quality of the referral service in Punjab, the primary data on users and non-users of 108 along with the data on physical inspection of 21 ambulances was analyzed. We used propensity score matched (PSM) non-users for each user (Table 1). Propensity score is the conditional probability of an individual to utilize 108 referral service from the group of users and non-users individuals taken together in the presence of covariates [15]–[17].

**Table 1:** Overview of Study Methodology for Evaluation of Emergency Referral Services in Punjab State, India.

S.No.	Evaluation Question	Data Source	Methodology	Indicator
1	Extent and pattern of utilization of the 108 emergency referral services	Secondary data for calls received during one year period (1^st^ April, 2012 to 31^st^ March 2013) in Punjab	Descriptive analysis	Call rate per ambulance per day and per ambulance per millon population per day.
				Mean number of ambulances per 1000 sq km, patients transported per day, km travelled per patient.
				Response to call
				Utilization pattern by type of Health facilities and emergency
2	Quality of service	Secondary data for calls received during one year period (1^st^ April, 2012 to 31^st^ March 2013) in Punjab	Propensity Score Matching	Response to call, by time of day
		Primary facility level data on users and non-users of 108 for districts Amritsar, Sangrur and Roopnagar	Means and Proportions	Time taken to reach health facility
		Ambulance inspection against BLS standards		Client satisfaction
				Proportion of calls attended
				Proportion of ambulances which meet the benchmarks for BLS
3	Impact of Referral Transport on Equitable utilization of public health facilities and Institutional Deliveries in Punjab	Primary facility level data on users and non-users of 108 for districts Amritsar, Sangrur and Roopnagar	Bivariate analysis	Proportion of users and non-users as per basic characteristics
		Public institution delivery data from 2008–2013	Multiple Logistic regression	Likelihood of equitable utilization
			Interrupted Time series (Segmented Linear regression)	Trend of institutional delivery, secular trend, intervention effect and change in trend
4	Unit health system cost of operating the ambulance services to the Punjab Government	Secondary data for calls received during one year period (1^st^ April, 2012 to 31^st^ March 2013) in Punjab	Cost analysis for recurrent and non-recurrent costs	Cost per call
		Secondary data on Operational and Non-operational costs		Cost per patient
				Cost per km travelled



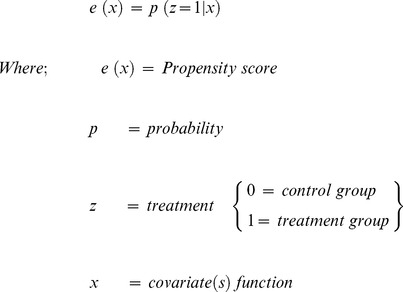



The regression analysis showed that the users and non-users of ERS were significantly different on a set of independent factors. Any comparison of time taken to reach the health facility from the time of emergency could thus be confounded by these factors. Other studies also show that these factors influence the different types of delays in accessing health facility for obstetric care [18], [19].

We used propensity score matching to generate a probability score for each individual (user or non-user) for using ERS, contingent upon a set of characteristics. The covariates which were included for generating propensity score included distance of place of emergency from health facility, locality of residence of the individual, education of head of household, socio-economic status of household, and district. Nearest neighbourhood method within permissible calliper of 0.02 (7% of the mean propensity score) was used. Overall the matched sample comprised of 276 pairs of users and non-users. Mean time taken to reach the health facility among users and matched non-users was computed. We analysed the effectiveness of ERS on reducing the 2^nd^ delay of the 3-delay model of Kwast, which means the time taken since the decision is made to seek medical care till the pregnant woman reaches health facility [20]–[23]. Additionally, client satisfaction among 108 users and proportion of calls attended for users by the time of the day (derived from calls data) were computed as quality measures of the referral transport system. Physical quality of ambulance was measured against BLS benchmarks (Table 1).

#### Impact of Referral Service on Public Sector Institutional Delivery

Trends in public institution deliveries before and after the introduction of 108 referral service were assessed (Table 1). It was assumed that intervention would start displaying its effect after the first phase itself when 90 ambulances (out of 240 planned) were already active for service delivery. Interrupted time series analysis was undertaken using Segmented Linear Regression (SLR) [24]. SLR works on the equation given below;



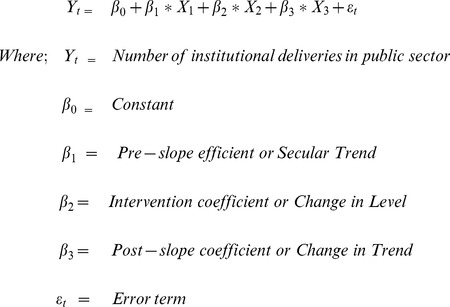



Presence of autocorrelation was examined upto 12^th^ lag using Durbin Watson test statistics and autocorrelation and partial autocorrelation functions [25]. The coefficients for secular trend (β_1_), change in the level of public institutional deliveries after the intervention was introduced (β_2_) and trend of the public institutional delivery in post-intervention period (β_3_) were estimated along with their 95% confidence intervals. Best fitting linear model was obtained after adjusting for autocorrelation and seasonal variation [24]. A sensitivity analysis was done with varying assumptions of time of treatment effect i.e. after first, second, third and fourth phase of implementation. Four prediction models were tested which differed based on the assumption of showing treatment effect at different time points. In the base model, it was assumed that the intervention shows its effect after first phase (April 2011). In the 2nd and 3rd model, it was assume that the intervention shows its effect after second phase (July 2011) and fourth phase (November 2011) respectively. In the final model, it was assumed that the intervention shows its effect 6 months after the complete implementation of the service.

The crude birth rate per 1000 population declined from 17.6 in 2007 to 16.2 in 2011 [26]–[30]. This could have potentially confounded the analysis. In order to test this, we used ‘proportion of deliveries which occurred in public sector institutions’ (out of total pregnancies) instead of total monthly public sector institutional deliveries as the dependent variable in SLR. Using the annual figures on births, population and growth rate, month-wise estimations for total population and births in Punjab was done using best fitting exponential curve. Numbers of pregnancies were estimated using an assumption of 10% pregnancy wastage. Finally, the proportion of pregnant women delivering in public institutions of Punjab was estimated using public institutions delivery data and total estimated pregnancies.

We examined the dose-response relationship by estimating correlation between the “proportion of deliveries where 108 service was used” in a district (indicative of ERS performance) with the district level “beta coefficient of impact of intervention” (indicative of impact of ERS on institutional delivery).

Costing was done from a health system perspective. The analysis of cost incurred on the referral service was based on broad classification i.e. capital and recurrent costs (Table 1). We annualised the costs of all the capital items based on the average life span of each item and discounting the costs at 3%. Equivalent uniform annual costs for each capital item were computed. We used cost paid to private provider per ambulance during 2012 as operational cost. Unit cost was estimated as cost per call, cost per individual transported and cost per kilometre travelled.

### Ethical Considerations

The study was approved by the Institute Ethics Committee of the Post Graduate Institute of Medical Education and Research, Chandigarh, India. Administrative approvals were obtained from the Health Department of Punjab state. Written approval was obtained from Civil Surgeons of the concerned districts and officer in-charge of health facilities which were visited. Written informed consent was also obtained from all study subjects (patients and staff) who were interviewed.

## Results

### Extent and Pattern of utilization of ERS

The density of ambulance was 4.8 per 1000 square km, with variation from 8.2 in Mohali to 2.8 in Sangrur and Roopnagar. The average number of calls received per ambulance per day was 4.5, and patients transported per ambulance per day was 3.5 (Table 2). In more than three-fourths (78%) calls, service was availed. Almost 37% of calls were received during the morning hours (8AM-2 PM) with 32% and 31% between 2PM to 8PM and during night hours (8PM-8AM) respectively. Majority (87.3%) of the ERS users were transported to public healthcare institutions, were females (70%), and had maternal and perinatal complications (76%).

**Table 2:** Performance Characteristics of Referral Transport services in Punjab, 2012–13.

	Characteristics
District	No. of Ambulance	Mean no. of ambulances per 1000 sq km	Mean no. of calls per ambulance per day	Mean no. of calls per lac population per day	Mean no. of patients transported per ambulance per day	Mean distance travelled per ambulance per day
**Amritsar**	22	4.3	4.7	4.2	3.7	64.5
**Barnala**	5	3.4	4.6	3.9	2.6	64.3
**Bhatinda**	12	3.6	4.5	3.9	3.5	73.9
**Faridkot**	5	3.4	5.6	4.5	8.9	72.6
**Fatehgarh Sahib**	5	4.2	3.9	3.3	3.0	79.2
**Firozepur**	17	2.9	4.7	4.0	2.5	63.4
**Gurdaspur**	20	5.6	4.1	3.5	3.1	66.5
**Hoshiarpur**	14	4.2	4.2	3.8	3.3	64.5
**Jalandhar**	19	7.1	4.2	3.7	3.3	60.7
**Kapurthala**	7	4.3	4.6	3.9	3.6	66.2
**Ludhiana**	30	8.0	4.4	3.8	3.5	58.7
**Moga**	7	3.2	5.1	4.7	5.3	69.4
**Mansa**	9	5.4	5.1	4.7	3.9	69.6
**Muktsar**	8	3.1	4.2	3.7	3.7	67.2
**Nawanshahar**	5	4.0	4.8	3.9	3.9	69.1
**Patiala**	16	4.4	4.3	3.7	3.7	84.2
**Roopnagar**	6	2.8	4.4	3.9	3.2	64.9
**Mohali**	14	2.8	3.9	3.3	2.3	60.9
**Sangrur**	9	8.2	4.8	4.4	4.0	74.9
**Tarn Taran**	10	4.1	4.4	3.9	3.5	78.6
**Total**	**240**	**4.8**	**4.5**	**3.9**	**3.5**	**67.9**

As per the locality, 60% and 54% of users and non-users belonged to rural area respectively (Table 3). Adjusting for other confounders, individuals at farther distance from the health facility and those belonging to poor socio-economic status were more likely to utilize the 108 referral service, thus implying an equitable ERS utilization (Table 3).

**Table 3:** Pattern of Referral Transport Service utilization in Punjab, India.

Characteristics		User	Non user	p-value	Odds Ratio* (95% Confidence Interval)			
					Model 1	Model 2	Model 3	Model 4
Gender	Male	127 (31)	367 (36.7)	0.04	Ref.	Not included in model	Not included in model	Not included in model
	Female	283 (69)	632 (63.3)		1.3 (0.8,1.5)			
Age	<5 yrs	11 (2.7)	25 (2.5)	<0.001	Ref.	Not included in model	Not included in model	Not included in model
	5–18 yrs	9 (2.2)	61 (6.1)		0.2* (0.06,0.8)			
	18–30 yrs	279 (68.2)	466 (46.6)		1.2 (0.5,2.7)			
	30–50 yrs	68 (16.6)	280 (28.0)		0.6 (0.2,1.4)			
	>50 yrs	42 (10.3)	167 (16.7)		0.6 (0.2,1.5)			
Residence	Urban	62 (15.0)	252 (25.2)	<0.001	Ref.	Ref.	Ref.	Not included in model
	Peri-urban	64 (15.6)	181 (18.1)		0.9 (0.6,1.5)	0.1 (0.6,1.6)	0.1 (0.6,1.6)	
	Slum	8 (1.9)	21 (2.1)		1.6 (0.6,4.2)	1.5 (0.6,3.8)	1.5 (0.6,3.7)	
	Rural	246 (59.8)	535 (53.5)		1.2 (0.8,1.7)	1.2 (0.8,1.8)	1.1 (0.8,1.7)	
Caste	Schedule Caste/Schedule Tribe	83 (20.2)	158 (15.8)	0.001	Not included in model	Not included in model	Not included in model	Not included in model
	Backward Class	36 (8.8)	100 (10.0)					
	Other Backward Class	49 (11.9)	144 (14.4)					
	General	156 (38.0)	445 (44.5)					
	Others	12 (2.9)	42 (4.2)					
Educational status of head of household	Illiterate	60 (15.3)	172 (17.8)	0.08	Ref.	Ref.	Not included in model	Ref.
	Primary	29 (7.4)	76 (7.9)		0.8 (0.4,1.4)	0.8 (0.4,1.5)		0.9 (0.5,1.6)
	Middle	57 (14.6)	159 (16.5)		0.9 (0.5,1.5)	0.1 (0.6,1.6)		0.1 (0.6,1.6)
	Matric	140 (35.8)	302 (31.3)		1.1 (0.7, 1.8)	1.2 (0.8,1.9)		1.3 (0.9,2.0)
	Secondary	87 (22.2)	180 (18.6)		1.5 (0.9,2.4)	1.6* (1.02,2.6)		1.7* (1.0,2.6)
	Graduate and above	18 (4.6)	76 (7.9)		0.9 (0.4,1.9)	1.1 (0.5,2.2)		1.1 (0.6,2.2)
Monthly expenditure	<5000 Rs	110 (33.0)	171 (21.6)	<0.001	2.8*** (1.6,4.7)	3.1*** (1.9,5.1)	2.7*** (1.7,4.3)	0.5*** (0.4,0.7)
	5000–10000 Rs	185 (55.6)	482 (60.8)		1.6* (1.0,2.6)	1.7* (1.1,2.6)	1.5** (1.0,2.4)	0.4*** (0.2,0.6)
	>10000 Rs	38 (11.4)	140 (17.7)		Ref.	Ref.	Ref.	Ref.
Distance of emergency place form health facility	1–5 kms	85 (20.9)	375 (37.7)	<0.001	Ref.	Ref.	Ref.	Ref.
	5–10 kms	108 (26.5)	200 (20.1)		2.1** (1.4,3.2)	2.2*** (1.4,3.3)	2.2*** (1.5,3.4)	2.3*** (1.6,3.4)
	10–15 kms	99 (24.3)	216 (21.7)		1.8** (1.2,2.7)	1.9** (1.3,2.9)	1.9** (1.3,2.9)	1.1*** (1.4,2.9)
	15–20 kms	55 (13.5)	116 (11.7)		2.0** (1.2,3.3)	2.2** (1.3,3.5)	2.2** (1.4,3.6)	2.4*** (1.5,3.8)
	>20 kms	60 (14.7)	87 (8.8)		2.9*** (1.8,4.8)	3.1*** (1.9,5.1)	3.3*** (2,5.3)	3.4*** (2.1,5.3)
Severity of emergency	1 (Most Severe)	5 (1.2)	2 (0.2)	0.05	Not included in model	Not included in model	Not included in model	Not included in model
	2	6 (1.5)	13 (1.3)					
	3	24 (5.8)	82 (8.2)					
	4	349 (84.9)	860 (86.1)					
	5 (Least Severe)	20 (4.9)	36 (3.6)					

*p<0.05, **p<0.01, ***p<0.001.

**Note 1**: Categories under caste i.e. *backward class, schedule caste and schedule tribe* is the standard nomenclature under the article 340, 341 and 342 respectively given in constitution of India. Categories under severity of emergency i.e. Severe = 1 and Normal = 5 represents maximum and minimum severity of medical emergency respectively.

**Note 2**: Outcome in all the models is use of 108 referral transport during any emergency and explanatory variables are given below;

**Model-1**: gender, age, residence of individual, educational status of head of household, monthly household expenditure, distance of place of emergency from health facility. **Model-2**: residence of individual, educational status of head of household, monthly household expenditure, distance of place of emergency from health facility. **Model-3**: residence of individual, monthly household expenditure, distance of place of emergency from health facility. **Model-4**: educational status of head of household, monthly household expenditure, distance of place of emergency from health facility.

### Impact of ERS on public sector institutional deliveries

The average number of deliveries in public sector institutions per month almost doubled after the introduction of 108 referral service in Punjab (5717 in pre-intervention and 10173 in post-intervention). An average of 3167 deliveries occurred in public sector institutions per month in the state before the intervention. There was a significant increase in month-wise number of deliveries before intervention with a pre-slope coefficient (β_1_) value of 134 (95% CI; 85,183). On the other hand, there was an insignificant change after intervention with a post-slope coefficient (β_3_) value of −22.3 (−105.1, 60.5). Model 1 suggests that there was sudden and significant increase in number of deliveries by 2341 (95% CI; 705, 3978) deliveries per month immediately after the introduction of 108 service in April 2011. It reflects the abrupt increase in the rate of public sector institutional deliveries occurred after the introduction of 108 referral service in Punjab (Table 4).

**Table 4:** Impact of referral transport service on public sector institutional deliveries in Punjab, April 2008-June 2013.

		β (95% confidence Interval)		
Model (Unadjusted)	Constant	Intervention effect	Pre-slope	Post-slope
**1**	3167***	2341.5** (704.9,3978)	134.2*** (85.4,182.9)	−22.3 (−105.1,60.5)
**2**	3127***	2979.1*** (1410.5,4547.7)	136.7*** (96.3,177.2)	−99.3* (−192.1,−6.5)
**3**	2541***	−550.1*** (−2359.6,1259.2)	177.7*** (139.4,215.9)	10.5* (−124.3,145.3)
**4**	2765***	−297.9 (−2306,1710.1)	163.8*** (129.6,197.9)	−34.7 (−230.4,160.9)
**Model 1 (Adjusted for first order autocorrelation)**	382	849.7 (−345.1,2044.6)	−14.5 (−50.1,21.1)	−41.4 (−101.9,19)
**Model 1 (Adjusted for seasonality)**	1386	1117.5 (−478.8,2713.7)	7.7 (−67.5,82.9)	−119.8** (−194.9,−44.6)
**Model 2 (Adjusted for first order autocorrelation)**	96.4	−454.6 (−1697,787)	6.7 (−25.3,21.1)	0.928 (−72.6,74.4)
**Model 2 (Adjusted for seasonality)**	1666*	1997 (486,3508)*	−6.1 (−65.7,53.5)	−184.6 (−269,−100.2)***
**Model 3 (Adjusted for first order autocorrelation)**	178	−486 (−1803,831)	1.4 (−26.4,29.2)	15.4 (−82.7,113.5)
**Model 3 (Adjusted for seasonality)**	593	−863 (−2502,777)	43.3 (−7.8,94.4)	−101 (−219,15.3)
**Model 4 (Adjusted for first order autocorrelation)**	321	550 (−886,1987)	−7.6 (−32,16.7)	−54 (−194,86)
**Model 4 (Adjusted for seasonality)**	636	−2983 (−4618,−1348)**	41.6 (1.8,81.5)*	57.6 (−97,212)

*p<0.05, **p<0.01, ***p<0.001.

**Note 1**: 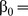 Constant, 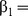
Pre-slope efficient or Secular Trend, 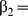
Intervention coefficient or Change in Level, 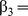
Post-slope coefficient or Change in Trend.

**Note 2**: Outcome in all the models is month-wise number of public institutional deliveries; model 1–4 differ based on the assumption of showing treatment effect at different time points. **Model-1**: Intervention shows its effect after first phase (April 2011). **Model-2**: Intervention shows its effect after second phase (July 2011). **Model-3**: Intervention shows its effect after fourth (last) phase (November 2011). **Model-4**: Intervention shows its effect 6 months after the complete implementation of the service.

**Figure 1:**
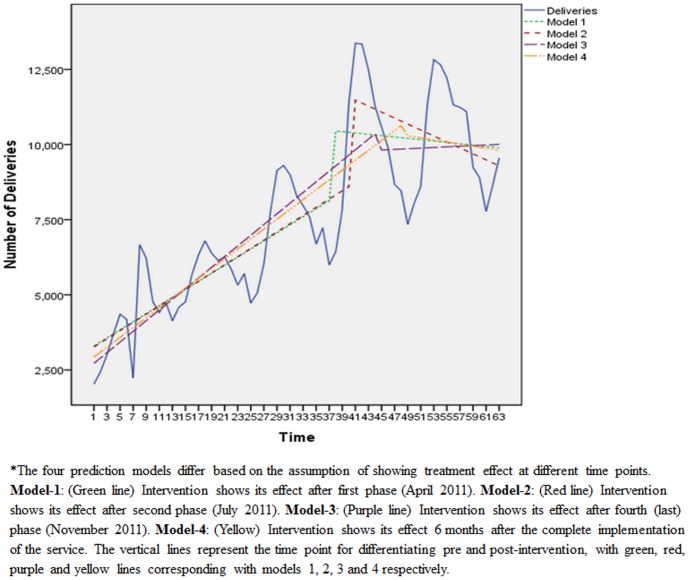
Actual trend and predicted trends of month-wise number of deliveries in public institution in Punjab, April 2008-June 2013.

The data series was adjusted for first order autocorrelation and seasonality. After adjustment for autocorrelation, we found no effect of ERS on institutional delivery. This reflects that the increase in institutional deliveries as a result of other interventions in the pre-intervention period could have had a secular trend which was showing an increase in institutional deliveries in the unadjusted model. (Table 4 and Figure 1).

We also adjusted for declining birth rate which could be a potential confounder. Estimated ‘monthly proportion of deliveries in public sector institutions’ out of total monthly estimated pregnancies was used as the dependent variable in SLR model. Results of this model validated our previous findings with a significant pre-slope and intervention effect but an insignificant post-slope. However, in this analysis also, adjustment of autocorrelation showed that the intervention has no effect on institutional delivery.

A dose-response relationship was tested, by correlating the extent of impact on public sector institutional deliveries at district level with utilization levels of ERS in the district. However, we found no significant dose-response relationship (r^2^ = 0.0064), thus confirming absence of any direct positive sustained effect of referral service on institutional delivery.

Findings of the sensitivity analyses were similar to base model, with ERS having an insignificant effect on public sector institutional deliveries in Models 2, 3 and 4 (Table 4 and Figure 1).

### Quality of ERS

**Figure 2:**
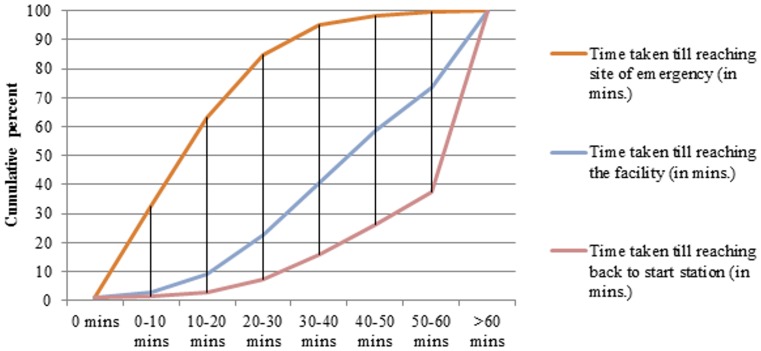
Timeliness of 108 referral transport service in Punjab, April 2012-March 2013.

In about one-third of calls, the ambulance reached the emergency site before 10 minutes, and within 20–30 minutes for 85% of calls. The time taken to transport patient to health facility was less than half hour among 23% cases and less than one hour in 74% cases. Average time taken by ERS to reach the emergency site and to transport the patient to health facility was 17.5 minutes and 48 minutes respectively (Figure 2). Among 78% of the callers, service was provided irrespective of time of call (day or night hours).

**Figure 3:**
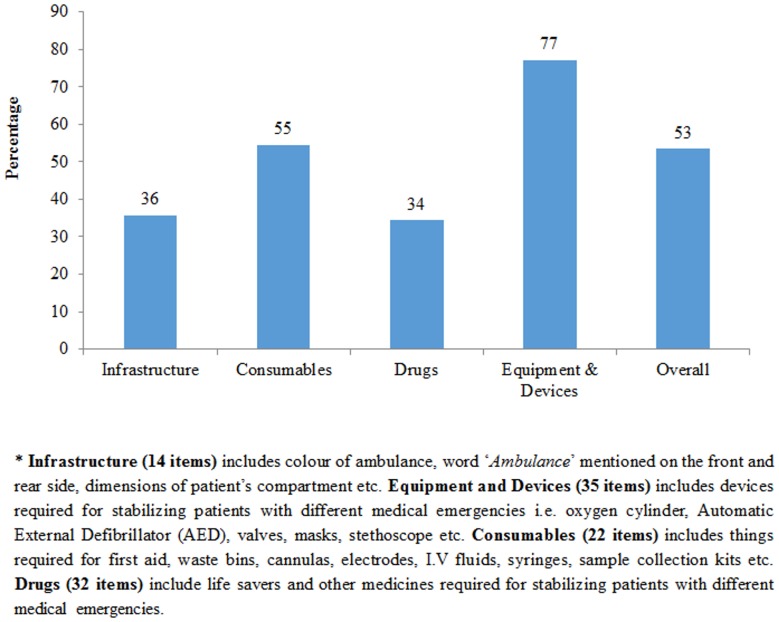
Quality of 108 Basic Life Support (BLS) Ambulances in Punjab.

The mean time taken to reach the facility by the user of 108 and propensity score-matched non-users was almost similar i.e. 26 minutes. Out of the 411 users, almost 90% rated ERS as ‘good’ or ‘excellent’, 6.3% as ‘average’ and 2.7% were dissatisfied with the service. Among 999 non-users, 89.4% were aware about the 108 service. Only 28 (2.8%) tried to call ERS, of whom in 10 cases call did not connect, while call could not be completed in 7 cases. In 5 cases, the call was completed but service denied, while remaining 6 did not give any reason for not availing the service. Out of the 21 ambulances assessed for infrastructure present against BLS standards, only 53% met the standards of Basic Life Support ambulance (Figure 3). Around 36% of standards for infrastructure i.e. colour of ambulance, word ‘Ambulance’ mentioned on the front and rear side, dimensions of patient's compartment etc. were met in ambulances. As against the recommended BLS guideline, about 55% consumables, 34% drugs and 77% of the equipment were available.

### Cost of ERS

In 2012, the total cost of operating ERS in Punjab was INR 448 (USD 7.5) million with an average annual cost of INR 1.87 (USD 0.03) million per ambulance. The estimated cost per call, cost per person transported and cost per kilometre travelled for referral service in Punjab was INR 1065 (USD 17.8), INR 1361 (USD 22.7) and INR 21 (USD 0.35) respectively. The cost per kilometre travelled ranged from INR 40 (USD 0.67) in district SAS Mohali to INR 9 (USD 0.15) in Faridkot district.

## Discussion

Strengthening of ERS has emerged as a major strategy for improving institutional care at delivery under India's flagship program - National Rural Health Mission [31]. A number of models have evolved in different states, primarily of two board nature- publicly financed and delivered, and publicly financed but privately delivered. We evaluated the latter model in Punjab state of India. Our study shows that the referral service is adequately being utilized in all districts with nearly 3–4 patients being transported per ambulance per day. This rate of utilization is reported to be optimal from efficiency viewpoint in an earlier evaluation [12]. Poor and those at a site away from the health facility have a higher likelihood of using the 108 service, reflecting equitable utilization. However, our results indicate that the Punjab ERS did not result in a significant increase on institutional deliveries in public sector institutions. Finally, the cost of operating the ERS service using a private provider is INR 1361 (USD 22.7) per patient transported or INR 21 (USD 0.35) per km travelled.

To our knowledge, no previous study has so far comprehensively evaluated the public-private referral transport service model in India. Some evaluations with focussed outcomes have been reported [32]. We evaluated all the aspects of the service i.e. utilization, effectiveness, quality and cost. We used established analytical methods and study designs to answer the study questions. Interrupted Time Series (ITS) designs are considered as robust methods to study intervention effects in a non-randomized setting. Unlike cross-sectional design comparing outcomes between groups at a single point of time and pre-post designs which merely compare estimates at two time point, ITS allows to control for existing trends and study the dynamics of intervention effect improving the validity of results [33]. ITS design allows for the statistical investigation of potential biases in the estimate of the effect of the intervention. These potential biases include secular trend – the outcome may be increasing or decreasing with time before the intervention; cyclical or seasonal effects – there may be cyclical patterns in the outcome that occur over time; or duration of the intervention – the intervention might have an effect for the first three months only after it was introduced and in such case yearly data would not have identified this effect; and random fluctuations – these are short fluctuations with no discernible pattern that can bias intervention effect estimates [34]. Another major problem could be the presence of autocorrelation, i.e. the errors (or residuals) of the fitted model are correlated with each other at particular time lags.

In order to circumvent these biases in causal attribution of intervention effect, segmented linear regression (SLR) analysis and Auto-Regressive Integrated Moving Average (ARIMA) modelling based on the Box–Jenkins methodology using interrupted time series data have been recommended [34]. Lagarde (2012) recommends SLR over ARIMA, grading former technique better on the basis of less data requirement, and having an explanatory approach than predictive [24]. Hence we believe that our choice of statistical modelling methods is well justified. We also found evidence of autocorrelation which we controlled in the model. Our data sources were also robust. Extent and pattern of utilization was assessed using secondary data of almost 0.4 million calls received for a one year period. This large volume of data resulted in robust findings, without any seasonal confounding. Since the payment to private operator for ERS is contingent upon a minimum usage of ambulance, it is very unlikely that there would be underreporting of the data. Further, since it is linked to a call which is made by the user, it is again unlikely that it could be over-reported.

The second important data which we used is that on institutional deliveries and births in public sector facilities in the state of Punjab which was obtained from Punjab Health Systems Resource Centre, which is part of the Department of Health. Any possibility of a change (increase or decrease) in the coverage of registration of deliveries and births in public sector facilities in Punjab is bound to confound our analysis. The birth registration in India increased from 59% to 83% between 2002 and 2011 respectively. In Punjab, the level of registration was 89% in 2002 and has been 100% since 2004 till 2011. Similarly, the registration of deaths in Punjab has been more than 90% since 2004. This implies that the data of vital events such as births or institutional deliveries used by us has been universal and constant since 2004 and is unlikely to confound the results. Similarly, time series data was also available for almost 36 months before and 27 months after intervention. We adjusted for possible confounding as a result of declining birth rate and the role of autocorrelation using differencing method [35].

Absence of control area to assess the impact on institutional delivery was the major limitation of our study. As the 108 service was implemented in all the 22 districts of Punjab, there was no scope of including control area in the study. However, we addressed this limitation by examining a dose response relationship. We observed no dose-response relationship (r^2^ = 0.0064), thus confirming absence of any direct positive sustained effect of referral service on institutional delivery. Our data did not permit us to examine the difference in the third delay and quality of care received between women arriving with an ambulance and those with private transport or no transport. This is recommended as a potential area for future research.

The major confounders for effectiveness analysis of ERS on institutional deliveries could be conditional cash transfer scheme for institutional delivery (Janani Suraksha Yojana, JSY), introduction of village-level Accredited Social Health Activist (ASHA) for generating community demand for institutional delivery care, strengthening of Primary Health Centres for 24×7services, and Janani Shishu Suraksha Karyakram (JSSK) to provide cashless deliveries in public sector health facilities. The former 3 strategies were implemented during the period from 2005 to 2007 and continued thereafter. These interventions are likely to influence the pre-trends during pre-intervention period of our analysis, and potentially lead to a secular trend before the intervention. The final intervention, JSSK, was launched in early 2012 which could potentially confound the effect of intervention by affecting the post-slope of our analysis. We adjusted for first-order autocorrelation which removed the secular effect of the first three confounding interventions (JSY, ASHA and 24×7 PHC strengthening). Given the context and data limitations, it was not possible to remove the effect of JSSK from intervention effect. However, since there is insignificant effect on institutional deliveries in post-intervention period, we can conclude that ERS and JSSK did not have a positive impact on the institutional delivery coverage in public sector health facilities in Punjab.

An increase in institutional deliveries in public sector can happen as a result of two factors. Firstly, it can arise by conversion of home deliveries to institutional deliveries. This was examined in the main analysis, where we found no impact. Secondly, it can occur by shifting the utilization of services from private to public sector. We tested this in a separate analysis, where the dependent variable was proportion of public sector deliveries among the total institutional deliveries in Punjab state. This analysis also showed statistically insignificant change, implying that the ERS did not result in a shift in utilization of delivery care from private to public sector.

Overall, literature suggests mixed results of strengthening referral transport systems on utilization of health care services [36]. The impact is context specific, dependent on organizational and structural factors. We conclude that from Indian context, ERS is likely to have a positive impact on institutional deliveries in areas where the lack of physical access is a major factor for not utilizing institutional care at the time of delivery [32], [37]–[39]. However, in states like Punjab, which have high baseline coverage of institutional delivery [40], good network of roads and health facilities which are close to community, high per capita income and high literacy level, other strategies for reducing maternal mortality are likely to be more cost effective rather than ERS.

The average number of ambulance per million population is 8.7 in Punjab, which is quite close to states like Andhra Pradesh, Gujarat and Karnataka implementing this service in a PPP mode. Mean number of patients transported per ambulance per day (3.5) in Punjab is also quite similar to Andhra Pradesh (3.59). The mean cost per patient transported was higher in Punjab as compared to Andhra Pradesh (INR 565; USD 9.4). The total cost of ERS service for the year 2012 was almost 6.2% of the total National Rural Health Mission budget of the state of Punjab. In other states with private provision of ERS, proportion of NRHM budget spent on paying the private provider varied from 3.1% in Rajasthan and 5.3% in Karnataka for the year 2012–13 [41]. Thus the cost of operating ERS in Punjab is higher than privately provided ERS models in other Indian states. The average time taken by ambulance, since its dispatch till patient transported to health facility, for those states where referral services are provided by Emergency Management Research Institute (EMRI) was 54 minutes [10]. This was similar to the time taken in Punjab (48 minutes).

To conclude, our study shows that the utilization of ERS in Punjab was adequate. Moreover, it was equitably utilized by the economically and geographically disadvantaged. However, the ERS did not have any significant effect on public sector institutional deliveries. This implies that provision of ERS to reduce demand-side barriers to utilization of institutional obstetric care is not the panacea to improve institutional deliveries. It has to be equally and comprehensively supported by efforts towards increasing the capacity of health system, health centres and hospitals, for provision of basic and emergency obstetric care. The effect on institutional deliveries is likely to be context specific and dependent on the baseline rates of institutional delivery. Lastly, the ERS service in Punjab needs to be strengthened in terms of reduction of cost and improvement in quality.
